# Structural Integrity of the Greek Key Motif in βγ-Crystallins Is Vital for Central Eye Lens Transparency

**DOI:** 10.1371/journal.pone.0070336

**Published:** 2013-08-06

**Authors:** Venkata Pulla Rao Vendra, Garima Agarwal, Sushil Chandani, Venu Talla, Narayanaswamy Srinivasan, Dorairajan Balasubramanian

**Affiliations:** 1 Hyderabad Eye Research Foundation, L. V. Prasad Eye Institute, Hyderabad, India; 2 Molecular Biophysics Unit, Indian Institute of Science, Bangalore, India; 3 Novarus Discoveries Pvt Ltd, Hyderabad, India; 4 University of Miami Miller School of Medicine, Miami, Florida, United States of America; University of Arkansas for Medical Sciences, United States of America

## Abstract

**Background:**

We highlight an unrecognized physiological role for the Greek key motif, an evolutionarily conserved super-secondary structural topology of the βγ-crystallins. These proteins constitute the bulk of the human eye lens, packed at very high concentrations in a compact, globular, short-range order, generating transparency. Congenital cataract (affecting 400,000 newborns yearly worldwide), associated with 54 mutations in βγ-crystallins, occurs in two major phenotypes nuclear cataract, which blocks the central visual axis, hampering the development of the growing eye and demanding earliest intervention, and the milder peripheral progressive cataract where surgery can wait. In order to understand this phenotypic dichotomy at the molecular level, we have studied the structural and aggregation features of representative mutations.

**Methods:**

Wild type and several representative mutant proteins were cloned, expressed and purified and their secondary and tertiary structural details, as well as structural stability, were compared in solution, using spectroscopy. Their tendencies to aggregate *in vitro* and *in cellulo* were also compared. In addition, we analyzed their structural differences by molecular modeling *in silico*.

**Results:**

Based on their properties, mutants are seen to fall into two classes. Mutants A36P, L45PL54P, R140X, and G165fs display lowered solubility and structural stability, expose several buried residues to the surface, aggregate *in vitro* and *in cellulo,* and disturb/distort the Greek key motif. And they are associated with nuclear cataract. In contrast, mutants P24T and R77S, associated with peripheral cataract, behave quite similar to the wild type molecule, and do not affect the Greek key topology.

**Conclusion:**

When a mutation distorts even one of the four Greek key motifs, the protein readily self-aggregates and precipitates, consistent with the phenotype of nuclear cataract, while mutations not affecting the motif display ‘native state aggregation’, leading to peripheral cataract, thus offering a protein structural rationale for the cataract phenotypic dichotomy “distort motif, lose central vision”.

## Introduction

Cataract, or the opacification of the eye lens, is the leading cause of blindness the world over. Since the eye lens is effectively a protein-packed elastic gel, with practically no molecular turnover with time, cataract is often regarded as a ‘protein disorder disease’. While age-related cataract results from the accumulation of environmental and metabolic effects, congenital cataract, seen in newborn children, is essentially genetic in etiology. It is the latter that we focus our attention in this report. Congenital cataract is an important cause of childhood blindness, affecting about 400,000 newborns worldwide yearly [1].

Mutations in 36 genetic loci and over 22 genes have been reported to be associated with congenital cataracts in humans [2]–[4]. Of these, the major genes are those of the crystallins, a class of cytosolic proteins that constitute 95% of the water-soluble structural proteins, contributing to about 35% of the lens mass. Of the three groups of crystallins, two members of the α-family- αA and αB, account for 30%, seven of the β-family (βA1, βA2, βA3, βA4 and βB1, B2 and βB3) for about 35% and three γ-crystallins (γC, γD and γS) about 25% of the total crystallin content in the human lens. And the distribution of the various crystallins within the lens is asymmetric and biphasic [5]). Lens fiber cells, which constitute the nuclear zones and bulk of the lens, are richer in βγ-crystallins than in the cortex and epithelial cells [6], [7].

While the crystal structures of native multimeric α-crystallins are currently being investigated [8], structural analysis in solution suggests the monomers to be largely in the β-pleated sheet conformation and a globular tertiary structure [9], [10]. The crystal structures of several β- and γ-crystallins are seen to be folded using a superfamily termed the βγ-crystallin fold- a double domain structure containing a series of four highly stable “Greek key” motif [11]–[13]. The Greek key motif is an evolutionarily conserved super-secondary protein structural fold that offers structural compactness and high intrinsic stability against stress. How this Greek key-derived dense packing of the β- and γ- crystallins in the eye lens translates into transparency is an issue of biological functional interest. It is possible to address this issue by studying the molecular genetic analysis of congenital cataracts associated with mutations in human β- and γ- crystallins, since (a) these are Mendelian or monogenic disorders [2], and (b) the detailed molecular structures of these homologous proteins are available.

Twenty eight naturally occurring mutations in human γC-, γ D- and γ S-crystallins are reported to date, associated with congenital cataracts (see Table S1 in File S1). These mutations are associated with an interesting phenotypic dichotomy. About half of these 28 generate nuclear cataract which requires as early a clinical intervention as possible. Congenital bilateral nuclear opacity, which appears to be the most common autosomal dominant inherited form of cataracts [14], blocks the central visual axis and causes complications such as nystagmus and developmental amblyopia in the growing infant [15]–[17], and pediatric ophthalmologists need to intervene at the earliest [18], [19]. On the other hand, cortical and other types of peripheral cataracts do not demand early action, since they do not block the visual axis. We have, in this report, attempted to analyze the molecular phenotype of these mutations, i.e., analyze the changes in the properties of the normal or wild type protein brought about by the mutation and how these relate to the pathology.

We start our study of the protein structural rationale behind this phenotypic dichotomy by concentrating on human γD-crystallin as the representative, since (a) 17 of the 28 mutations in human γ-crystallins are seen in this molecule, and (b) there is remarkable structural homology among the various γ-crystallins. Human γD-crystallin (HGDC) exists largely as a β-pleated sheet, folded in four Greek key motifs, in a double domain structure. Motif 1 covers the sequence 1–40, motif 2 is between residues 42–83, motif 3 is in the sequence 88–128 while the last Greek key is found in the stretch 129–171. Its structure, both in the crystal and solution states, is well known [20]–[24]. Likewise, the structural analysis of several of its single point mutants, namely R14C, P24T, R37S (all present in the first Greek key motif), W43R, R58H, R77S (all in Greek key 2) and E107A (in Greek key 3), have been done in detail [25]–[37].(Note: The amino acid residues in several publications are numbered counting the N-terminal starting methionine residue as 1 (e. g. P24T, R77S), while other publications discount met 1 and count residues as (P23T,R76S). In order to avoid confusion and maintain uniformity, we number residues here, counting methionine as met-1).

Interestingly, none of these above mutations affect the Greek key topology in any significant manner,and they all are mostly associated with peripheral cataracts. On the other hand, other mutations that we study here, e. g., A36P (distorting the first Greek key motif) and Y134X, R140X, W157X and G165fs (each of which disturbs the fourth Greek key motif through truncation of the chain or frame shift). And all these mutations are associated with nuclear cataract. We have cloned, expressed and isolated each of these proteins and compared their structural and aggregation properties with those of the wild type. We have also revisited P24T, R77S and E107A and collected some more relevant data for comparison. In addition, we have prepared two (not reported in nature) full length chain mutants: Y134A (which is a mutation in the fourth Greek key motif, but one that still keeps all the 4 motifs intact, which has also been prepared and studied contemporaneously by Kong and King [24]) and the double mutant L45PL54P (which disrupts the second Greek key motif in the N-terminal half), and compared their properties with the others. Based on these results, we extend our discussion to the reported mutations in other γ- and β-crystallins present in the human infant lens (Tables S1 in File S1).

## Results

### Solubility and Conformational Features in Solution

Substantial differences are observed in the solubility of the various mutants. Wild type HGDC is highly soluble in water (400 mg/ml), and all the mutants display lower solubility- E107A to the extent of 325 mg/ml [37], and some others (P24T, R77S), while quite soluble, tend to start aggregating beyond 30 mg/ml [38]. The mutant A36P was found to be soluble only up to 5 mg/ml and precipitates beyond that. Several others such as Y134A, R140X, and G165fs are far less soluble, <200 µg/ml; indeed these sparingly soluble molecules came out in the inclusion body when they were cloned, expressed and isolated. We had found earlier that the solubility of the mutant W157X to be <250 µg/ml [39].

**Figure 1:**
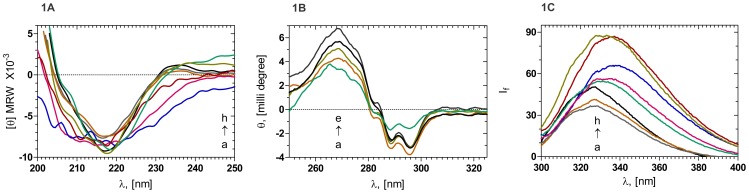
A: Secondary structural features of HGDC and its mutants. Mean residue molar ellipticities, in degrees, of A (Blue): G165fs; B (Violet): L45PL54P; C (Maroon): R140X; D (Brown): R77S; E (Grey): P24T; F (Black): WT; G (Olive): A36P; and H (Green): Y134A. Protein concentration in the case of WT, P24T, R77S, and A36P was 12 µM and in the case of R140X, G165fs, L45PL54P and Y134A it was 6 µM in 50 mM Tris buffer (pH 7.3). MRW = mean residue molecular weight, taken as 110 Da. The cell path length was 2 mm and all spectra were recorded at 37°C, corrected for background buffer signal and each reported spectrum is an average of 3 independent runs. **B: Tertiary structural features of HGDC and its mutants.** Ellipticities, in millidegrees, of A (Green): Y134A; B (Brown): R77S; C (Olive): A36P; D (Black): WT; and E (Green): P24T. Protein concentration in each case was 24 µM 50 mM Tris buffer (pH 7.3) and cell path length 10 mm, while the other conditions were maintained same as above. **C: Intrinsic fluorescence of HGDC and mutants.** I_f_: emission intensity in arbitrary units. A (Grey): P24T; B (Brown): R77S; C (Black): WT; D Y134A; E (Violet): L45PL54P; F (Blue): G165fs; G (Maroon): R140X; and H (Olive): A36P: λ_exc_: 295 nm, cell path length 3 mm, excitation and emission slits 2.5 nm recorded at room temperature. Each reported spectrum is an average of 3 independent runs.

Figures 1A and 1B compare the ultraviolet circular dichroism (CD) spectral features of the wild type and the various mutant human γD-crystallins. Only minor changes are seen to occur between the spectra of the mutants in comparison to that of the wild type, suggesting that the overall conformation is not altered in any significant manner upon mutation. Figure 1C monitors the tertiary structural features of the proteins around their aromatic side chains, through the intrinsic fluorescence spectra in the 300–400 nm region. The emission maximum of wild type (WT) molecule is seen to occur at 327.5 nm, with a relative intensity of emission (I_f_) of 50.5 arbitrary units, in agreement with earlier reports. Those of P24T and R77S are seen to be at 328.0 (I_f_ 37) and 327.0 nm (I_f_ 41), respectively. In comparison, the mutant Y134A displayed a slightly red-shifted intrinsic emission at 333 nm (I_f_ 55), while A36P emits at 333 nm, and R140X, G165fs and the N-td double mutant L45PL54P had their bands around 337 nm with intensities of 85, 65 and 55, respectively. These suggest that the aromatic side chains in these mutants that affect the Greek key motif are somewhat more exposed to the solvent than in the others. This was supported by experiments using the ionic quencher KI which quenches the emission of Trp and Tyr residues which are surface-accessible [40]. The Stern-Volmer quenching constants of the KI quenching varied in the order A36P> Y134A>>R77S >P24T >WT; the solutions of the other mutants precipitated upon KI addition.

**Figure 2:**
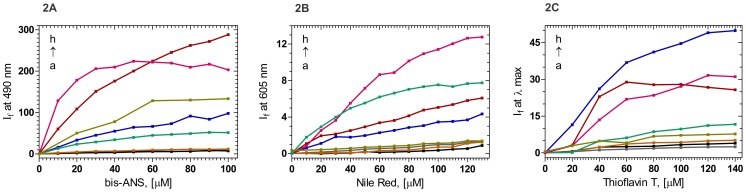
A:Surface exposure of residues in the proteins, monitored using bis-ANS as the extrinsic probe. A (Black): WT; B (Grey): P24T; C (Brown): R77S; D (Green):Y134A; E (Blue): G165fs; F (Olive): A36P; G (Violet): L45PL54P; and H (Maroon): R140X. I_f_ at 490 nm of the probe was measured as a function of its increasing concentration λ_exc_: 390 nm, cell path length 3 mm, excitation and emission slits 2.5 nm. Each curve is an average of 3 independent runs. **B: Aggregation tendencies of the proteins, estimated using Nile Red as the extrinsic probe.** A (Black): WT; B (Brown): R77S; C (Grey): P24T; D (Olive): A36P; E (Blue): G165fs; F (Maroon): R140X; G (Green): Y134A; and H (Violet): L45PL54P. I_f_ at 605 nm of the probe was measured as a function of its increasing concentration. λ_exc_: 540 nm, cell path length 3 mm, excitation emission slits 10 nm. Each curve is an average of 3 independent runs. **C: Using Thioflavin-T to probe amyloid-type aggregation of HGDC and its mutants.** A (Grey): P24T; B (Black): WT; C (Brown): R77S; D(Olive): A36P; E (Green): Y134A; F (Maroon): R140X; G (Violet): L45PL54P; and H (Blue): G165fs. I_f_ of the probe at λ_max_ was measured as a function of increasing concentration. Protein concentration in each case was fixed at 6 µM, cell path length 3 mm, excitation and emission slits 5 nm. Each curve is an average of 3 independent runs.

A better way of monitoring such solvent exposure of buried residues is through the use of extrinsic fluorophores such as bis-ANS, which bind to the surface of the protein and, upon so binding, show enhanced emission intensity [41]. Figure 2A shows that while WT and the mutants P24T and R77S generate little or no extrinsic fluorescence of the added bis-ANS, the other mutants do, and in the order R140X>L45PL54P> A36P> G165fs>>Y134A> the rest. Another probe that is used as an extrinsic fluorophore is the neutral dye Nile Red, which is also used to assess the tendency of the host protein to self-aggregate [42]. Figure 2B shows that when it is added to solutions of each of the HGDC mutants, its extrinsic fluorescence intensity at 605 nm varies in the order L45PL54P> Y134A>R140X>G165fs>A36P> the rest. Here again, a clear difference is noticed between the mutants that disturb the Greek key folding and those that do not. (Note that Y134A,despite its secondary structural similarity with WT, P24T and R77S,tends to aggregate; it is not clear whether this is due to fact that the region V126-Y134 in HGDC is suggested to have an “intrinsic aggregation propensity” [43]).

### Chemical and Thermal Stability of the Mutants

**Figure 3:**
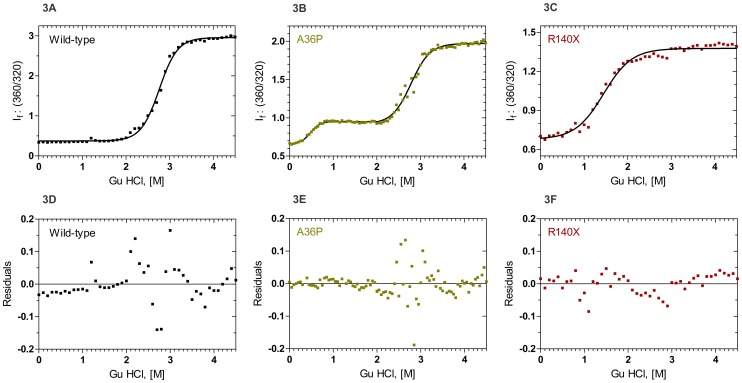
Guanidine hydrochloride (GuHCl) induced denaturation of wild type and mutant γD crystallins. Samples were excited at 295 nm and the relative emission intensity of the 360 nm band (of the denatured form) was compared to that of the 320 nm band (of the native protein) and monitored as a function of denaturant concentration. Solid line indicates the fitted data and solid blocks stand for raw data. Protein concentration in each sample was fixed at 0.2 mg/ml in 50 mM Tris buffer, 1 mM EDTA and 5 mM DTT. Residuals of wild type and mutant are also shown below the graphs.

Figure 3 shows the chemical denaturation profiles of WT, A36P and R140X mutants of HGDC, at room temperature using the denaturant guanidine hydrochloride (GuHCl). The WT molecule denatures in a single step with the midpoint of transition at 2.81 M GuHCl (defined as the Cm value), and the free energy ΔG° value, estimated using the two-state-denaturation model [44], of 7.76 kcal mol^−1^. On the other hand, the mutant A36P displays an intermediate state of unfolding, with a midpoint (C_m1_) around 0.45 M GuHCl before it unfolds totally with a midpoint (C_m2_) value of 2.73 M GuHCl. Using the three-state denaturation model [45], we estimate the apparent ΔG°_1_ value of 2.61 kcal mol^−1^ for the first transition and ΔG°_2_ value of 7.61 kcal mol^−1^ for the second transition. Such three-state transitions have been reported earlier for some other mutants of HGDC (24), and with G18V and V42M mutants of human γS-crystallin [46]–[48]. The truncation mutant R140X appears to undergo a simple two-state unfolding, with a Cm value of 1.83 M GuHCl and a ΔG° value of 2.65 kcal mol^−1^. This is reminiscent of what Kong and King [24] found for the N-terminal domain alone of HGDC (ΔG° 3.7 kcal mol^−1^ and Cm 1.21 M GuHCl).The loss of 4^th^ Greek key appears to make R140X behave somewhat similar to the N-terminal domain of HGDC, possibly because it might not display a strong inter-domain contact due to chain truncation. The chemical denaturation profiles of R77S, E107A and P24T have been reported by others [35], [37], [38], and they all follow the simple two-state model of unfolding, though each of them denatures at slightly lower GuHCl concentrations (<0.01 M GuHCl) than the WT. And we were not able to study the denaturation of L45PL54P and G165fs due to solubility problems.

We next used differential scanning calorimetry in order to study the thermal denaturation profiles of the molecules. Using a protein concentration of 0.6 mg/ml (about 24 µM) and scanning from 25°C up to 95°C at the rate of 1°C/min, we obtained a thermal melting temperature (Tm value) of 81.5°C for the wild type, 78.9°C for P24T, 82.5°C for R77S, and 78.5°C for E107A. In contrast, the mutant A36P displayed a Tm value of 48.5°C (but the protein started precipitating after 55°C, and hence we had to stop collecting data after 60°C). And since the mutants L45PL54P, R140X and G165fs started precipitating upon raising the temperature beyond 40°C, we could not estimate their Tm values.

### Nature of the Protein Aggregates

That γ-crystallins form amyloid type filaments when they are unfolded *in vitro*, or present in a particular mutant form in mice lenses has been reported [49]–[51]. Keeping this in mind, we monitored this tendency with the aggregates formed by all the mutants. A ready method to check this is the use of the dye thioflavin T, which upon binding to an amyloid-forming protein, displays a significant redshift and enhanced intensity in its emission band in the 470–570 nm region [52]. Figure 2C compares the effect of the various mutants of HGDC on the emission properties of thioflavin T, under conditions of room temperature, near neutral pH, and incubation for about 30 min. Here again, the effect is in the order: G165fs>L45PL54P> R140X>>>Y134A>A36P> R77S = P24T = wt, suggesting that the aggregates in the former four cases might have some amyloid-type character.

### In situ Studies: Mutant Protein Aggregates in Cells

**Figure 4:**
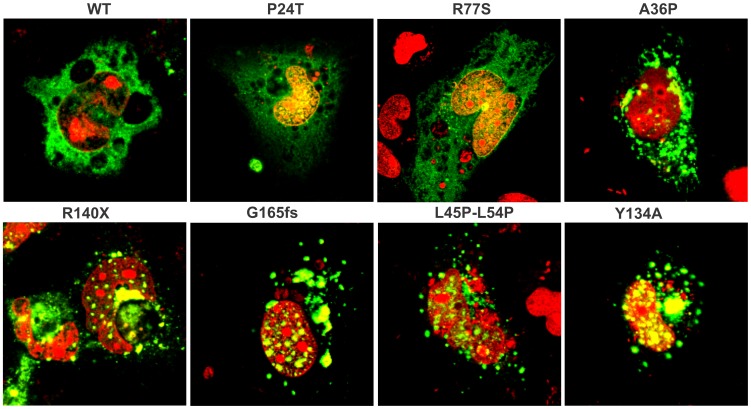
Visualizing the aggregation of the protein *in situ* in the human lens epithelial cell line HLE-3B using confocal microscopy. In each case, 6- His-tagged cDNA of the protein was transfected and visualized using anti- His antibody and FITC-conjugated secondary antibody. The nuclei of the cells were visualized using propidium iodide; Magnification: 630X.

Figure 4 shows the confocal microscopic images of human lens epithelial cells HLE 3B transfected with wild type and the various mutant cDNAs, tagged with 6X His tag and probed with (mouse) anti-His antibody, and FITC-conjugated anti-mouse secondary antibody. The nuclei of the cells were counterstained with propidium iodide and visualized in red color. The figure shows that unlike the wild type, P24T and R77S, which are distributed uniformly across the cell, A36P, L45PL54P, R140X and G165fs form punctate particles, suggesting *in situ* aggregation. We had earlier shown a similar *in situ* formation of light scattering particles when the other two C-terminal mutants E107A [36], and W157X [39] were likewise transfected. (Mutant Y134A too shows some scattering particles, perhaps due to the report that the sequence V126-Y134 in the molecule has an intrinsic propensity for aggregation [43]). It is, however, not clear whether the aggregates are self-aggregates or mixed aggregates, as was established in the case of E107A [37].

## Discussion

### Surface Exposure of Residues Upon Mutation

The high resolution crystal structure of human γD- crystallin [20] makes it possible to model the wild type, as well as its mutants. We have used the interactive graphics software SETOR [53] to understand the consequences of each mutation on the structure of the molecule, including the accessible surface areas of exposed residues [54]. The figures have been rendered using PyMOL [55], and Table 1 gives a summary of the structural analysis of the wild type and various mutants of HGDC, including the solvent exposure of relevant residues, possible inter-domain interactions, and other features.

**Table 1:** Summary of the structural analysis of various mutants of human γD crystallin.

S.No.	Mutants	Secondary structure and solvent accessibility of the residue	Non polar residues with increased solvent exposure in the mutant*	Polar residues with increased solvent exposure in the mutant*	Effect on Inter-domain interactions	Intermolecular interactions at the site of mutation
1	P24T	Edge strand in the second Greek key (GK) motif in the N-terminal domain is solvent-exposed	Y6,Y16,Y45,Y50,Y62, A63,Y98,F118,W157,A159	T4,E7,R9,Q12,R14,H15,E17,D21,P23),S30,N33,R36,D38,Y45,Q47,S51,R59,D61,H65Q66,Q67,S72,D73,R76,S87,H88,R89,R95,R99,Q101,C111,Q113,R115,N125,E128,R140,T160,R163	Indirectly	Not directly affected
	A36P	GK-1motif disturb due to Proline	P43,L53, F118,F173	R9,N33, C41,Q47, H65,Q66, D 97,R99,C111,	GK1 not involved in interdomain interactions. However, the conformation change and destabilization of GK1 might indirectly affect.	Altered stability and conformation of the GK1 might affect Intermolecular interactions
3	L45PL54P	Residues are in the middle and edge strands, respectively, in GK 2. L45 is buried, L54 is solvent-exposed	L53, M69, L71,	R9, E46, D64, Q66, Q67, S74, R76, R99	GK2 involved in domain-domain interactions. The conformation and stability of this β sheet is affected due to the presence of prolines	Altered stability and conformation of the GK2 might affect Intermolecular interactions
4	R77S	One of the middle strands in GK motif 2 in N-terminal domain is solvent -exposed	Y55, F118, I171	R9, F24, Q26, N33, E46,Q47,N49,Q66,Q67,S72,S74,S77,D97,R99,Q101	No	Occurs close to a positively charged patch in symmetry- related molecule
5	E107A	Loop connecting the two GKs in C-terminal domain, solvent-exposed	L53,F105,F118	R9,N33,Q66,R76,H88,R89, D97,R99,Q101,T106,D108,C109,R169	No	Polar environment in the symmetry- related molecule
6	Y134A	Located at the middle strand in GK4, buried	L53, M69, F118, V132, Y139,	R9,N33, E46, D64, N66, N67, S74, R76, D97, R99, Q101,R140, Q143	Possibly affected as the site occurs in GK4 involved in inter-domain interactions	Does not seem to be affected
7	R140X	Three strands corresponding to both the motifs and a loop connecting the two strands	L53,F56,I81,I90,L92,Y98,L112,I121,L124,V126,L127,W131,V132,L133,Y134,L136,	Y45,Q54,R59,R79,S84,H88,E96,D97,Y98,T106,D108,C109,Q113,N119,E120,H122,N125,S130,E135,S137,N138, *#Y144,L145,L146,W157,A159,A162,V164,L167,V170*	Affected	Could be affected due to unfolding of the molecule
8	W157X	Loop and beta strand at the C-terminal region	F56,I81,L92,L112,I121,L124,W131,V132,L133,Y134,L136,Y139,Y144,L146,Y151,Y154,	Q54,R59,S84,H88,T106,D108,C109,Q113,N119,E120,S130,E135,S137,R142,Y144,Y154, *#W157,A159,A162,V164,L167,V170*	Affected	Could be affected due to unfolding of the molecule
9	G165fs		L53,F56,I81,L92,F118,W131,V132,L133,Y139,	R9,N33,R59,Q66,Q67,R76,S84,H88,D97,R99,Q101,T106,D108,C109,S130,Y134,*#V170*	Affected	Possibly affected due to unfolding

# The residues indicated in italics are the residues buried in the WT but absent in the respective mutants.

**Figure 5:**
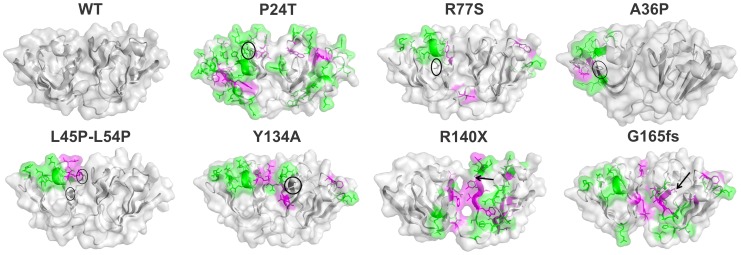
Modeled structures of HGDC and mutants. The residues with increased nonpolar surface exposed to the solvent are indicated in magenta, and polar residues indicated in green; the site of mutation is highlighted in black.

Figure 5 shows the modelled structures of the various mutants, where we show residues contributing to the increased nonpolar surface in the mutant in magenta color, and those contributing to increased polar surface in green. Looking first at the mutant P24T, the Thr residue at the site of mutation has been labelled. Exposure of numerous nonpolar residues might reduce the solubility of the molecule. Increased exposure of polar residues,as also from Cys 111 (labelled in the figure), would predispose the protein to aggregation.

In the mutant A36P, the backbone φ angle at the site of mutation in the WT is -156.4°. As the residue Pro constrains φ to -60°, drastic alteration in the backbone conformation is expected, thus disrupting the tertiary structure as well. Additionally, we find that the coulombic interaction that A36 has with surface D residues of the WT molecule is lost upon replacing A36 by P. The exposure of nonpolar residues P43, L53, F118 amd F173 (see Table 1) would also encourage aggregation.

On the other hand, in the double mutant L45PL54P, the Greek key motif 2 is disrupted, affecting the conformation and inter-domain interactions. Interestingly, more polar residues than nonpolar ones are exposed (see Table 1). When we next look at R77S, we find that mutation of Arg 77 to Ser reduces the solvent exposure as well as the positive charge at the site. The altered surface potential as well as the exposure of nonpolar groups might affect its association with other protein molecules and reduce its solubility.

The mutation Y134A is located in the middle strand of the 4^th^ motif, and while it does not disrupt the topology, it does expose as many as 5 apolar and 13 polar side chains, which would lead to decreased solubility and possible aggregation.

In the mutant R140X, we note that a significant fraction of the region contributing to the C-terminal domain is lost upon truncation. The deleted region includes three β-strands which form an integral part of the jellyroll fold of the C-terminal domain. Loss of three β-strands and five buried apolar residues in the deleted region is expected to result in the near complete loss of structure of the C-terminal domain. Significant loss of tertiary structure, greater exposure of nonpolar residues and of Cys 109 would be expected to lead to aggregation of the molecule. With the other truncation mutant W157X too, the situation is quite the same, as seen in Figure 5, and in Table 1.

We believe this would also be the case with the other mutant, Y134X, reported to occur in a Danish infant with congenital cataract-microcornea syndrome; in comparison to R140X, this mutant has lost the hexapeptide sequence ELSNYR, comprising almost all surface-seeking residues. The situation with Y56X, which has lost even greater part of the chain, is expected to be far more severe (see Table S1 in File S1 for phenotypic details of these mutations).

The frameshift mutation in G165fs results in premature termination, resulting in the loss of a C-terminal β-strand. The exposure of Cys 109 and of multiple nonpolar residues along with the loss of tertiary contacts due to the missing β-strand would affect the stability of the molecule.

### Other Members of the γ-crystallin Family

While we have described our analysis of mutations in HGDC so far, we find it interesting to note that the reported mutations in other human γ-crystallins (γC, γS) and even β-crystallins display a similar phenotypic dichotomy. We briefly review these below. Table S1 in File S1 also lists the mutations reported in human γC- and γS- crystallins and the associated cataract types. Here again we notice the nuclear-peripheral dichotomy. The N-terminal domain mutants T5P and Gfs62 (neither disturbing the Greek key motif ) in human γC- crystallin are associated with a Coppock-type cataract and zonular pulverulant cataract, respectively, while C109X, S119S (actually a polymorphism), and W157X (all of which disturb the Greek key fold) generate nuclear cataracts. R168W, which does not affect the Greek key, causes lamellar cataract. Turning to human γS-crystallin, four mutations in this molecule have so far been reported, all in the N-terminal domain, i.e., G18V, D26G, S39C and V42M. Neither G18V [46], [47] nor D26G (our own unpublished results) alter the motif and are associated with peripheral cataracts, as also S39C though its structural analysis is not reported yet. V42M is reported to be associated with dense cataract; our molecular and structural analysis [51] shows how the mutation distorts the second Greek key motif.

### Mutations in Human β-crystallins

Since the folding patterns of β- and γ-crystallins are quite similar, we have analyzed the results of 27 congenital cataract mutations (Table S2 in File S1) reported to date in the members of the human β-crystallin family, along with the relevant references and comments therein. We briefly review them here. β -crystallin differs from γ-crystallin in that it has an N-terminal extension of 15 residues, the domain 17–56 forming the first Greek key, 57–101 the second, 107–148 the third and 149–191 the fourth Greek key motif, and a longer linker region of 65 residues between the N- and C-terminal domain regions of the molecule. This longer linker leads to intermolecular aggregation of β -crystallin through the binding of the N–td of one molecule with the C-td of a second one, leading to a multimeric native structure. (In γ- the shorter linker region forces an intra-molecular interaction of the C-td with the N-td, leaving the molecule to be folded as a stable monomer). Interestingly, here too we notice the putative connection between the structural intergrity of the Greek key motif and and phenotypic dichotomy. Note that 7 of the 8 mutations reported in βB1-crystallin, associated with nuclear cataracts, are C-terminal domain mutants which disturb the Greek key motifs 3 or 4. The mutation M1K abrogates the initiation codon and generates a nonsense non-crystallin molecule. Turning to human βB2-crystallin, mutations A2V, I21N and S31W do not affect any of the Greek key motifs, while the others would be predicted to, and lead to, nuclear cataracts. In βB3, R75H affects the second motif and destroys a highly conserved R75, while G165R is reported to open the hairpin fold and destabilize the fourth Greek key motif, and is associated with nuclear cataract.

Turning to human βA3/A1-crystallin, the first 2 exons encode the amino terminal arm, and exons 3–6 encode Greek keys 1–4. Most mutations here predominantly lead to exon skipping and thus lose sequences containing one or more Greek key motifs, and are invariably associated with nuclear cataract. The others reported follow a pattern similar what has been seen above with the γ-crystallins.

With βA4-crystallin, 3 mutations are reported, all on exon 4, and these impair the beta strands forming the Greek key, destabilizing the protein by as much as 5 kcal/mol. All these lead to nuclear cataract, accompanied by microcornea/microphthalmia.

### Caveats

There are some caveats that, however, need to be pointed out. One is that protein aggregation can also arise from intermolecular disulfide cross-linking, as reflected in mutants R14C and G61C of human γD-crystallin, both of which lead to dense particles radiating from the center of the lens. Mutant W59C of βB2 might also do likewise. The other is the formation of hetero-molecular aggregates through Coulombic interactions (or effects of altered pI values upon mutation), e. g., E107A of HGDC which engages in complexation with the acidic partner α-crystallin. It is likely that W43R, which too is reported to generate nuclear and perinuclear cataract, might display a similar charge-driven intermolecular aggregate, since its estimated pI is about 7.65 (cf 7.0 for the wild type). Likewise, R48H of human γC-crystallin, and mutant I21N in βB2, associated with nuclear cataracts, might aggregate hetero-molecularly through Coulombic forces. The other caveat is: we are not considering mutations in other genes, e.g., α-crystallins and connexins, which too lead to nuclear cataract, but these proteins do not fold using the Greek key motif topology.

### Congenital Cataracts in Mice

While our discussion so far has been on human cataracts, many similar congenital cataracts have been reported in mice [56]. Though there are differences in the length and actual sequences between mouse and human crystallins, there is considerable sequence homology between them. In addition, while the human lens expresses essentially only γC, γD and γS, mice lenses express γA, γB, γC, γD and γE crystallins. Even here mutations that affect the Greek key folding (or alter the pI of the mutant significantly, e.g., W43R γA, V76D γD, D77G γA, W168R βA1) seem to be associated with nuclear cataracts.

Our results, and analysis of all mutations reported so far, suggest that when the mutation disrupts even one of the Greek key folds in the βγ- crystallin family, the resultant protein (a) unfolds, exposing several buried residues to the surface, (b) becomes sparingly soluble in water, (c) tends to generate amyloidogenic aggregates *in vitro*, and (d) displays light scattering particles when transfected in cell lines, all of which are consistent with the phenotype of nuclear cataract. These mutations lead to what has been termed as “protein disorder disease”. In contrast, mutations that do not disturb the Greek key motifs maintain the overall chain folding and only produce local disturbances around the mutation site leading to peripheral cataracts due to reduction in solubility; this situation has recently been described as one involving well-folded proteins but with aberrant homologous protein interactions [57] (also termed ‘native state aggregation’ [50], or ‘protein condensation disease’ [58], [59]. The structural integrity of the Greek key motif in βγ-crystallins thus appears to be an essential element in packing the crystallins in a compact, close-packed manner. Such packing appears to offer long term stability and stress resistance to the βγ-crystallins [60], and the short-range order they exhibit in the lens is thought to be responsible for the transparency of the lens [61].Our results suggest that disruption of even one of these motifs in the chain leads to nuclear cataract and central vision loss. “Lose Greek key, lose central vision”.

## Methods

Wild type and mutant γD crystallin genes were cloned into pET-21-a and p_C_DNA3.1(+) vector as previously described [36].

### Overexpression of Recombinant Proteins

The recombinant constructs pET21-a-γD wild-type, pET21-a-γDP24T, pET21-a-γDR77S, pET21-a-γDA36P, pET21-a-γDL45PL54P, pET21-a-γDY134A, pET21-a-γDR140X and pET21-a-γDG165fs were transformed into E. coli BL21(DE3) pLysS cells. A single colony containing the recombinant constructs was picked, inoculated into 15 ml of Luria-Bertoni (LB) medium containing 50 µg/ml ampicillin and 34 µg/ml chloramphenicol and grown for 8 hr by shaking at 225 rpm at 37°C. After 8 hr, 10 ml of the culture was transferred into 1L of LB medium containing 50 µg/ml ampicillin and 34 µg/ml chloramphenicol. The cultures were grown at 37°C to an absorbance value of 0.6 at 600 nm. Protein synthesis was induced by the addition of isopropyl-1-thio-D-galactopyranoside (IPTG) to a final concentration of 1 mM and the cultures were grown for an additional 3.5 h. Cells were pelleted down from the 1L culture by centrifugation at 6000 *g* for 10 min at 4°C.

The cell pellets were suspended in 40 mL of Lysis Buffer containing 50 mM Tris-Cl (pH 7.5), 100 mM KCl, 1 mM dithiothreitol (DTT), 1 mM phenylmethylsulfonyl fluoride (PMSF) and 20 µg/ml aprotinin. The cell suspension was extensively sonicated for 40 cycles (30 s/cycle) with 30 s intervals at 35% amplitude at 4°C using a high intensity ultrasonic processor (Sonics Vibra Cell, Sonics & Materials Inc, Newton, MA).The cell lysate was centrifuged at 30,000 *g* for 20 min at 4°C. The supernatant and the pellet were checked for the presence of the protein. Wild-type, P24T, R77S, Y134A, and A36P were predominantly found in the soluble fraction, whereas, L45PL54P, R140X and G165fs were found in the inclusion bodies. We overexpressed all these proteins at different IPTG concentrations (0.25, 0.5 and 1.0 mM), at various temperatures (18°C and 37°C) and various time points (2.5 h, 3.0 h and 3.5 h), but, in all these conditions, L45PL54P, R140X and G165fs were found insoluble and hence we had to purify the proteins from inclusion bodies.

### Purification of WT, P24T, R77S, Y134A and A36P Mutant Proteins

The supernatant was chromatographed using a Phenyl-Sepharose column. The fractions containing the required protein were pooled, concentrated and further purified to homogeneity by gel filtration chromatography using a Sephadex G-75 column. In the case of A36P and Y134A, the supernatant was directly loaded to G-75 column. Fractions containing the protein of interest were further purified using a SP-Sepharose column.

### Purification and Refolding of L45PL54P, R140X and G165fs Mutant Proteins

The insoluble pellet in each case was resuspended in 15 ml of washing buffer (25 mM Tris-Cl (pH 8.0), 100 mM KCl, 0.1% Triton X100, 1.5 M urea and 2 mM DTT) and washed 5–6 times by centrifugation at 30,000 *g*, for 20 min at 4°C until a clear supernatant was obtained. The pellet was then washed with 15 ml of wash buffer lacking urea and Triton X100 and dissolved in 40 ml of buffer containing 50 mM Tris (pH 7.3) and 7.5 M urea and stirred continuously to solubilize the protein and incubated at 4°C for 12 h.

We were not successful in refolding the denatured protein by On-column refolding using Q or SP Sepharose matrix and rapid dilution; hence we adopted the stepwise dialysis approach. The protein mixture was dialyzed initially against 50 mM Tris (pH 7.3) and 7.5 M urea under continuous stirring at 4°C. Next, 50 mM Tris buffer (pH 7.3) was added drop by drop till the protein completely loses urea. The purity of the proteins was assessed by SDS-PAGE, western blotting and MALDI mass spectral analysis. The concentration of each protein was measured by A280 in 4.5 M GuHCl, using its molar extinction coefficient calculated from Expasy http://web.expasy.org/cgi-bin/protparam/protparam. The estimated mass numbers of each protein obtained using MALDI mass spectral analysis were: WT: observed 20597 (expected 20610), P24T: 20615 (expected 20614), R77S: 20532 (expected 20540), A36P: 20580 (expected 20636), L45PL54P:20550 (expected 20578), Y134A: 20488 (expected 20518), R140X: 16448 (expected 16453) and G165fs: 19659 (expected 19662).

### Spectroscopic Analysis of Recombinant Proteins

Spectroscopic analysis of wild type and mutant proteins was done using circular dichroism and fluorescence emission spectroscopy. Extrinsic fluorescence of the proteins were recorded using bis-ANS and Nile Red respectively. The amyloid type behavior was monitored using Thioflavin T. The chemical stability of the proteins was assessed using guanidine hydrochloride, and the thermal stability was studied using differential scanning calorimetry as previously described [48].

### Cell Culture and Transfections

For *in vitro* experiments HLE-3B cells were used, which have been previously characterized [62]. These cells were cultured in Dulbecco’s Modified Eagle Medium (DMEM) (Sigma) supplemented with 20% fetal bovine serum (FBS) (HyClone) containing antibiotics in a 5% humidified CO_2_ incubator at 37°C. 12 hr prior to transfection, 1,50,000 cells were seeded on a 18 mm cover slip in a six-well culture plate and incubated in a 5% humidified CO_2_ incubator at 37°C. The complete medium was removed after 12 hr and replaced with antibiotic free medium and the cells were transfected with the recombinant constructs using Fugene HD (Promega) at a 1∶6 ratio (1 µg vector/6 µl lipofectamine). After incubation for 4 hr, one ml of complete medium was added and incubation was continued up to 24 hr for imaging. Immunofluorescence and Imaging was done as described in our earlier paper [36].

## Supporting Information

File S1 Table S1, Mutations reported in human γD-, γC- and γS-crystallins. Table S2, Mutations in Human β- Crystallins associated with congenital cataracts.
(DOC)
