# Supplementary material for: Structural Integrity of the Greek Key Motif in βγ-Crystallins Is Vital for Central Eye Lens Transparency
Source: PLoS One. 2013 Aug 6;8(8):e70336. doi: 10.1371/journal.pone.0070336 (PMC3735602; doi:10.1371/journal.pone.0070336)
Supplement: File S1 — Table S1, Mutations reported in human γD-, γC- and γS-crystallins. Table S2, Mutations in Human β- Crystallins associated with congenital cataracts. (DOC) [file pone.0070336.s001.doc]

**Supporting Information**

**Supplementary Table S1: Mutations reported in human γD-, γC- and γS-crystallins**

**Table S1A: Mutations reported in Human γD-Crystallin**

| **S.No** | **Mutation** | **Type of cataract seen** | **Reference** | **Comments** |
| --- | --- | --- | --- | --- |
| 1 | R14C | Nuclear and perinuclear | [1,2] | Only surface change; extensive disulfide  intermolecular bridges see (Ref.26 in main text) |
| 2 | R14S | Coralliform | [3] | Slight change in hydrophobic content, opening up a phosphorylation site (no conformational work) |
| 3 | P24T | Coralliform, Cerulean, Fasciculiform | [4-10] | Extensive conformational analysis done by many shows no change in sec/tert structure, minor surface changes, drop in solubility (see ref.27-31 in main text) |
| 4 | A36P | Nuclear | [11] | Greek key compactness distorted, higher hydrophobic exposure upon mutation; solubility drops. (see data in main text) |
| 5 | R36P | Nuclear opacity | [12] | No data yet |
| 6 | R36S | Birefringent crystals | [13] | Crystal structure reveals no changes. |
| 7 | W43R | Dominant congenital cataract | [14] | Minor tert struct. change; pI must be different, solubility not affected; (see ref.33 in main text) |
| 8 | M44V | Blue dot opacity | [15] | No structural data yet |
| 9 | Y56X | Nuclear | [16] | Large scale truncation of chain; three Greek key motifs lost |
| 10 | R58H | Aculeiform | [17] | H bonding lost. But no other change. pI altered |
| 11 | G61C | Coralliform | [18, 19] | No structural data; Disulfide polymers expected; hydrophobic content changes expected |
| 12 | R77S | Polar coronary cataract | [20] | No change seen in structure upon mutation. pI change? Solubility high (see ref.35 in main text) |
| 13 | E107A | Nuclear | [21] | Same as above. Solubility high. pI change is seen to lead to heteroaggregation with α-crystallin (see ref.37 in main text) |
| 14 | Y134X | Not reported | [22] | No data, but Greek key 4 gone |
| 15 | R140X | Nuclear | [23] | Greek key 4 lost; solubility lost; surface exposure extensive (see data in main text) |
| 16 | W157X | Nuclear | [24] | Greek key 4 gone; solubility lost; surface exposure extensive (see ref.39 in main text) |
| 17 | G165fs | Nuclear | [25] | Our data shows high hydrophobic exposure; solubility lost (see data in main text) |

**Table S1B: Mutations r**eported in Human γC-Crystallin

| **S.No** | **Mutation** | **Type of cataract seen** | **Reference** | **Comments** |
| --- | --- | --- | --- | --- |
| 1 | T5P | Coppock-like cataract | [17] | No data |
| 2 | G62fs | Zonular pulverulant | [26] | No data |
| 3 | C109X | Nuclear cataract | [27] | Truncation and loss of Greek key |
| 4 | S119S | Nuclear cataract | [16] |  |
| 5 | W157X | Nuclear cataract | [28] | Truncation mutant; data shows solubility loss, exposure of residues to surface (see ref.39 in main text) |
| 6 | R168W | Nuclear cataract | [23, 29] | pI change, as in E107A? (see ref.39 in main text) |
| 7 | R48H | Nuclear cataract | [30] | Similar to the gamma D- mutant |

**Table S1C: Mutations reported in Human γS-Crystallin**

| **S.No** | **Mutation** | **Type of cataract seen** | **Reference** | **Comments** |
| --- | --- | --- | --- | --- |
| 1 | G18V | Progressive cortical cataract | [31] | Extensive data shows no change in 2° or 3°, but compaction of GK 1 likely to alter (see ref.46,47 in main text) |
| 2 | D26G | Coppock cataract | [11] | Very little change in 2° or 3°, solubility high. (manuscript submitted) |
| 3 | S39C | Microcornea-cataract | [23] | No data, but disulfide polymers likely |
| 4 | V42M | Autosomal dominant cataract | [32] | Compact packing of Greek key is distorted.  (see ref.48 in main text) |

**Note-1:**Residue numbers in column 2 are as reported with cited references, regardless of whether met 1was counted or not.

**Note-2:** Coralliform: round or elongated processes radiating out of the center of the lens; Cerulean: small bluish dots; Fasciculiform: fibrous radiative strands emanating from the center; Aculeiform: frosted lens; Polar coronary: ring around a clear central lens, slowly progressive cortical opacity; Coppock-like: bilateral progressive opacity of the embryonic nucleus, pulverulent (fine powderish); Zonular/lamellar: affecting only certain layers between nucleus and cortex.

**Supplementary Table S2: Mutations in Human β- Crystallins associated with congenital cataracts**

**Table S2A: Mutations in Human β B1**- Crystallins associated with congenital cataracts

| **S. No** | **Mutation** | **Phenotype** | **Reference + comments** |
| --- | --- | --- | --- |
| 1 | Met 1 Lys MIK  (exon 2) | Bilateral, mild nuclear, pulverulant cataract | [33]*;* abrogates initiate codon, even if translation occurs, it loses greek key 1 and part of GK 2 |
| 2 | Del G 168 in  (exon 2),  i. e., N58T fs 106 | Bilateral nuclear  opacification | [34]; elongates the C-terminus, disrupting the β-crystallin interactions and truncation |
| 3 | S129R | Bilateral nuclear cataract and microcornea | [35, 36] Destabilizes the βB1/βA3-crystallin heteromer but not the βB1 homomer; also sensitizes βB1 to proteolysis |
| 4 | G220X | Pulverulant, affecting  fetal nucleus | [37]; truncation of 33% of the 4^th^ GK, and the entire C-terminus, losing solubility |
| 5 | Q 223X | Nuclear | [38]; truncation, losing part of 4th GK motif |
| 6 | S228P | Nuclear cataract | [39]*;* loss of the conserved S228, which, along with a distal gly, enables the supersecondary fold by packing the hairpin over the β-sheet in the GK motif. Replacing it by the structure breaker P disturbs the supersecondary fold. |
| 7 | R233H | Nuclear | [40]; PolyPhen analysis shows damage to the protein structure, and loss of solubility due to loss of the conserved cationic R. |
| 8 | X253R | Dense nuclear microcornea | [41]; elongates the C-terminal extension and disrupts β-crystallin interactions. |

**Table S2B:** Mutations in human βB2- crystallin and congenital cataracts in children

| **S.No.** | **Mutation** | **Phenotype** | **References** |
| --- | --- | --- | --- |
| 1 | A2V | Posterior subcapsular cataract | [42] |
| 2 | I21N | Dense white opacities throughout the embryonic and fetal nuclei | [40] |
| 3 | S31W | Coronary cataract | [43] |
| 4 | cDNA-G54A base pair exchange in last base of exon 2 | Zonular cataract | [15] |
| 5 | W59C | Central opacity in both lenses | [15] |
| 6 | D128V (mutation outside exon 6) | Nuclear in both eyes, with by ring-shaped opacity | [44] |
| 7 | V146M | Nuclear | [40] |
| 8 | W151C | Dominant central nuclear cataract | [20] |
| 9 | Q155X | Variable genotype, but predominantly nuclear, Coppock-like, pulverulant opacity in embryonic nucleus | [45-52] |
| 10 | Y159X | Not reported | [53] |
| 11 | V187M | Nuclear | [54] |

**Table S2C:** Mutations in Human βB3- Crystallin and congenital cataracts in children

| **S. No** | **Mutation** | **Phenotype** | **Reference + comments** |
| --- | --- | --- | --- |
| 1 | c.224G>A, i.e.,  R 75H in the protein | Microcornea cataract | [53]; mutation affects the 2^nd^ GK motif, and destroys a highly conserved amino acid R75. |
| 2 | G 165R | Nuclear cataract with cortical riders | [55]; mutation  destabilizes 4^th^ GK Motif, & increases electrostatic charge surface |

**Table S2D: Mutations in hu**man βA3/A1- crystallin and congenital cataracts in children

| **S. No** | **Mutation** | **Phenotype** | **Reference + comments** |
| --- | --- | --- | --- |
| 1 | Splice site mutation  in first base in intron 3: CryBA1-IVS3+1 G>A | Autosomal dominant nuclear, pulverulant, zonular, Y-sutural opacity: intra-pedigree variability | [24, 56-59]: exon skipping abolishes Greek Keys |
| 2 | Splice site mutation in first base in intron 3: CryBA1-IVS3+1 G>C | Autosomal dominant pulverulant opacities in embryonic nucleus, sutural opacities, | [60] |
| 3 | 3 bp deletion in exon 4 leading to deletion of gly 91; ∆91 | Autosomal dominant congenital nuclear cataract | [61-65]; defective folding of two β strands making the Greek Key motif |

**Table S2E:** Mutations in human βA4- crystallin and congenital cataracts in children

| **S. No** | **Mutation** | **Phenotype** | **Reference + comments** |
| --- | --- | --- | --- |
| 1 | F94S | Congenital lamellar cataract | [66] |
| 2 | L69P | Congenital lamellar cataract and microcornea, | [66] |
| 3 | G64W | congenital nuclear cataract  and microcornea | [67]; G64 is a highly conserved residue across species |

**References for the Tables of mutations**

1. Stephan DA, Gillanders E, Vanderveen D, Freas-Lutz D, Wistow G, et al. (1999) Progressive juvenile-onset punctate cataracts caused by mutation of the gammaD-crystallin gene. Proc Natl Acad Sci U S A 96: 1008-1012.
2. Gu F, Li R, Ma XX, Shi LS, Huang SZ, et al. (2006) A missense mutation in the gammaD-crystallin gene CRYGD associated with autosomal dominant congenital cataract in a Chinese family. Mol Vis 12: 26-31.
3. Zhang LY, Gong B, Tong JP, Fan DS, Chiang SW, et al. (2009) A novel gammaD-crystallin mutation causes mild changes in protein properties but leads to congenital coralliform cataract. Mol Vis 15: 1521-1529.
4. Hilal L, Nandrot E, Belmekki M, Chefchaouni M, El Bacha S, et al. (2002) Evidence of clinical and genetic heterogeneity in autosomal dominant congenital cerulean cataracts. Ophthalmic Genet 23: 199-208.
5. Nandrot E, Slingsby C, Basak A, Cherif-Chefchaouni M, Benazzouz B, et al. (2003) Gamma-D crystallin gene (CRYGD) mutation causes autosomal dominant congenital cerulean cataracts. J Med Genet 40: 262-267.
6. Mackay DS, Andley UP, Shiels A (2004) A missense mutation in the gammaD crystallin gene (CRYGD) associated with autosomal dominant "coral-like" cataract linked to chromosome 2q. Mol Vis 10: 155-162.
7. Xu WZ, Zheng S, Xu SJ, Huang W, Yao K, et al. (2004) Autosomal dominant coralliform cataract related to a missense mutation of the gammaD-crystallin gene. Chin Med J (Engl) 117: 727-732.
8. Shentu X, Yao K, Xu W, Zheng S, Hu S, et al. (2004) Special fasciculiform cataract caused by a mutation in the gammaD-crystallin gene. Mol Vis 10: 233-239.
9. Khan AO, Aldahmesh MA, Ghadhfan FE, Al-Mesfer S, Alkuraya FS (2009) Founder heterozygous P23T CRYGD mutation associated with cerulean (and coralliform) cataract in 2 Saudi families. Mol Vis 15: 1407-1411.
10. Yang G, Xiong C, Li S, Wang Y, Zhao J A (2011) recurrent mutation in CRYGD is associated with autosomal dominant congenital coralliform cataract in two unrelated Chinese families. Mol Vis 17: 1085-1089.
11. Sun W, Xiao X, Li S, Guo X, Zhang Q (2011) Mutation analysis of 12 genes in Chinese families with congenital cataracts. Mol Vis 17: 2197-2206.
12. Wang L, Chen X, Lu Y, Wu J, Yang B, et al. (2011) A novel mutation in gammaD-crystallin associated with autosomal dominant congenital cataract in a Chinese family. Mol Vis 17: 804-809.
13. Kmoch S, Brynda J, Asfaw B, Bezouska K, Novak P, et al. (2000) Link between a novel human gammaD-crystallin allele and a unique cataract phenotype explained by protein crystallography. Hum Mol Genet 9: 1779-1786.
14. Wang B, Yu C, Xi YB, Cai HC, Wang J, et al. (2010) A novel CRYGD mutation (p.Trp43Arg) causing autosomal dominant congenital cataract in a Chinese family. Hum Mutat 32: E1939-1947.
15. Santhiya ST, Kumar GS, Sudhakar P, Gupta N, Klopp N, et al. (2010) Molecular analysis of cataract families in India: new mutations in the CRYBB2 and GJA3 genes and rare polymorphisms. Mol Vis 16: 1837-1847.
16. Santana A, Waiswol M, Arcieri ES, Cabral de Vasconcellos JP, Barbosa de Melo M (2009) Mutation analysis of CRYAA, CRYGC, and CRYGD associated with autosomal dominant congenital cataract in Brazilian families. Mol Vis 15: 793-800.
17. Heon E, Priston M, Schorderet DF, Billingsley GD, Girard PO, et al. (1999) The gamma-crystallins and human cataracts: a puzzle made clearer. Am J Hum Genet 65: 1261-1267.
18. Li F, Wang S, Gao C, Liu S, Zhao B, et al. (2008) Mutation G61C in the CRYGD gene causing autosomal dominant congenital coralliform cataracts. Mol Vis 14: 378-386.
19. Zhang W, Cai HC, Li FF, Xi YB, Ma X, et al. (2011) The congenital cataract-linked G61C mutation destabilizes gammaD-crystallin and promotes non-native aggregation. PLoS One 6: e20564.
20. Roshan M, Vijaya PH, Lavanya GR, Shama PK, Santhiya ST, et al. (2010) A novel human CRYGD mutation in a juvenile autosomal dominant cataract. Mol Vis 16: 887-896.
21. Messina-Baas OM, Gonzalez-Huerta LM, Cuevas-Covarrubias SA (2006) Two affected siblings with nuclear cataract associated with a novel missense mutation in the CRYGD gene. Mol Vis 12: 995-1000.
22. Hansen L, Yao W, Eiberg H, Kjaer KW, Baggesen K, et al. (2007) Genetic heterogeneity in microcornea-cataract: five novel mutations in CRYAA, CRYGD, and GJA8. Invest Ophthalmol Vis Sci 48: 3937-3944.
23. Devi RR, Yao W, Vijayalakshmi P, Sergeev YV, Sundaresan P, et al. (2008) Crystallin gene mutations in Indian families with inherited pediatric cataract. Mol Vis 14: 1157-1170.
24. Santhiya ST, Shyam Manohar M, Rawlley D, Vijayalakshmi P, Namperumalsamy P, et al. (2002) Novel mutations in the gamma-crystallin genes cause autosomal dominant congenital cataracts. J Med Genet 39: 352-358.
25. Zhang LY, Yam GH, Fan DS, Tam PO, Lam DS, et al. (2007) A novel deletion variant of gammaD-crystallin responsible for congenital nuclear cataract. Mol Vis 13: 2096-2104.
26. Ren Z, Li A, Shastry BS, Padma T, Ayyagari R, et al. (2000) A 5-base insertion in the gammaC-crystallin gene is associated with autosomal dominant variable zonular pulverulent cataract. Hum Genet 106: 531-537.
27. Yao K, Jin C, Zhu N, Wang W, Wu R, et al. (2008) A nonsense mutation in CRYGC associated with autosomal dominant congenital nuclear cataract in a Chinese family. Mol Vis 14: 1272-1276.
28. Gonzalez-Huerta LM, Messina-Baas OM, Cuevas-Covarrubias SA (2007) A family with autosomal dominant primary congenital cataract associated with a CRYGC mutation: evidence of clinical heterogeneity. Mol Vis 13: 1333-1338.
29. Zhang L, Fu S, Ou Y, Zhao T, Su Y, et al. (2009) A novel nonsense mutation in CRYGC is associated with autosomal dominant congenital nuclear cataracts and microcornea. Mol Vis 15: 276-282.
30. Kumar M, Agarwal T, Khokhar S, Kaur P, Roy TS, et al. (2011) Mutation screening and genotype phenotype correlation of alpha-crystallin, gamma-crystallin and GJA8 gene in congenital cataract. Mol Vis 17: 693-707.
31. Sun H, Ma Z, Li Y, Liu B, Li Z, et al. (2005) Gamma-S crystallin gene (CRYGS) mutation causes dominant progressive cortical cataract in humans. J Med Genet 42: 706-710.
32. Vanita V, Singh JR, Singh D, Varon R, Sperling K (2009) Novel mutation in the gamma-S crystallin gene causing autosomal dominant cataract. Mol Vis 15: 476-481.
33. Meyer E, Rahman F, Owens J, Pasha S, Morgan NV, et al. (2009) Initiation codon mutation in betaB1-crystallin (CRYBB1) associated with autosomal recessive nuclear pulverulent cataract. Mol Vis 15: 1014-1019.
34. Cohen D, Bar-Yosef U, Levy J, Gradstein L, Belfair N, et al. (2007) Homozygous CRYBB1 deletion mutation underlies autosomal recessive congenital cataract. Invest Ophthalmol Vis Sci 48: 2208-2213.
35. Wang KJ, Wang S, Cao NQ, Yan YB, Zhu SQ (2011) A novel mutation in CRYBB1 associated with congenital cataract-microcornea syndrome: the p.Ser129Arg mutation destabilizes the betaB1/betaA3-crystallin heteromer but not the betaB1-crystallin homomer. Hum Mutat 32: E2050-2060.
36. Wang S, Zhao WJ, Liu H, Gong H, Yan YB (2013) Increasing betaB1-crystallin sensitivity to proteolysis caused by the congenital cataract-microcornea syndrome mutation S129R. Biochim Biophys Acta 1832: 302-311.
37. Mackay DS, Boskovska OB, Knopf HL, Lampi KJ, Shiels A (2002) A nonsense mutation in CRYBB1 associated with autosomal dominant cataract linked to human chromosome 22q. Am J Hum Genet 71: 1216-1221.
38. Yang J, Zhu Y, Gu F, He X, Cao Z, et al. (2008) A novel nonsense mutation in CRYBB1 associated with autosomal dominant congenital cataract. Mol Vis 14: 727-731.
39. Wang J, Ma X, Gu F, Liu NP, Hao XL, et al. (2007) A missense mutation S228P in the CRYBB1 gene causes autosomal dominant congenital cataract. Chin Med J (Engl) 120: 820-824.
40. Wang KJ, Wang BB, Zhang F, Zhao Y, Ma X, et al. (2011) Novel beta-crystallin gene mutations in Chinese families with nuclear cataracts. Arch Ophthalmol 129: 337-343.
41. Willoughby CE, Shafiq A, Ferrini W, Chan LL, Billingsley G, et al. (2005) CRYBB1 mutation associated with congenital cataract and microcornea. Mol Vis 11: 587-593.
42. Yao K, Li J, Jin C, Wang W, Zhu Y, et al. (2011) Characterization of a novel mutation in the CRYBB2 gene associated with autosomal dominant congenital posterior subcapsular cataract in a Chinese family. Mol Vis 17: 144-152.
43. Lou D, Tong JP, Zhang LY, Chiang SW, Lam DS, et al. (2009) A novel mutation in CRYBB2 responsible for inherited coronary cataract. Eye (Lond) 23: 1213-1220.
44. Pauli S, Soker T, Klopp N, Illig T, Engel W, et al. (2007) Mutation analysis in a German family identified a new cataract-causing allele in the CRYBB2 gene. Mol Vis 13: 962-967.
45. Litt M, Carrero-Valenzuela R, LaMorticella DM, Schultz DW, Mitchell TN, et al. (1997) Autosomal dominant cerulean cataract is associated with a chain termination mutation in the human beta-crystallin gene CRYBB2. Hum Mol Genet 6: 665-668.
46. Gill D, Klose R, Munier FL, McFadden M, Priston M, et al. (2000) Genetic heterogeneity of the Coppock-like cataract: a mutation in CRYBB2 on chromosome 22q11.2. Invest Ophthalmol Vis Sci 41: 159-165.
47. Vanita, Sarhadi V, Reis A, Jung M, Singh D, et al. (2001) A unique form of autosomal dominant cataract explained by gene conversion between beta-crystallin B2 and its pseudogene. J Med Genet 38: 392-396.
48. Yao K, Tang X, Shentu X, Wang K, Rao H, et al. (2005) Progressive polymorphic congenital cataract caused by a CRYBB2 mutation in a Chinese family. Mol Vis 11: 758-763.
49. Bateman JB, von-Bischhoffshaunsen FR, Richter L, Flodman P, Burch D, et al. (2007) Gene conversion mutation in crystallin, beta-B2 (CRYBB2) in a Chilean family with autosomal dominant cataract. Ophthalmology 114: 425-432.
50. Li FF, Zhu SQ, Wang SZ, Gao C, Huang SZ, et al. (2008) Nonsense mutation in the CRYBB2 gene causing autosomal dominant progressive polymorphic congenital coronary cataracts. Mol Vis 14: 750-755.
51. Liu BF, Liang JJ (2005) Interaction and biophysical properties of human lens Q155* betaB2-crystallin mutant. Mol Vis 11: 321-327.
52. Wang L, Lin H, Gu J, Su H, Huang S, et al. (2009) Autosomal-dominant cerulean cataract in a chinese family associated with gene conversion mutation in beta-B2-crystallin. Ophthalmic Res 41: 148-153.
53. Hansen L, Mikkelsen A, Nurnberg P, Nurnberg G, Anjum I, et al. (2009) Comprehensive mutational screening in a cohort of Danish families with hereditary congenital cataract. Invest Ophthalmol Vis Sci 50: 3291-3303.
54. Mothobi ME, Guo S, Liu Y, Chen Q, Yussuf AS, et al. (2009) Mutation analysis of congenital cataract in a Basotho family identified a new missense allele in CRYBB2. Mol Vis 15: 1470-1475.
55. Riazuddin SA, Yasmeen A, Yao W, Sergeev YV, Zhang Q, et al. (2005) Mutations in betaB3-crystallin associated with autosomal recessive cataract in two Pakistani families. Invest Ophthalmol Vis Sci 46: 2100-2106.
56. Kannabiran C, Rogan PK, Olmos L, Basti S, Rao GN, et al. (1998) Autosomal dominant zonular cataract with sutural opacities is associated with a splice mutation in the betaA3/A1-crystallin gene. Mol Vis 4: 21.
57. Burdon KP, Wirth MG, Mackey DA, Russell-Eggitt IM, Craig JE, et al. (2004) Investigation of crystallin genes in familial cataract, and report of two disease associated mutations. Br J Ophthalmol 88: 79-83.
58. Zhu Y, Shentu X, Wang W, Li J, Jin C, et al. (2010) A Chinese family with progressive childhood cataracts and IVS3+1G>A CRYBA3/A1 mutations. Mol Vis 16: 2347-2353.
59. Gu Z, Ji B, Wan C, He G, Zhang J, et al. (2010) A splice site mutation in CRYBA1/A3 causing autosomal dominant posterior polar cataract in a Chinese pedigree. Mol Vis 16: 154-160.
60. Bateman JB, Geyer DD, Flodman P, Johannes M, Sikela J, et al. (2000) A new betaA1-crystallin splice junction mutation in autosomal dominant cataract. Invest Ophthalmol Vis Sci 41: 3278-3285.
61. Reddy MA, Bateman OA, Chakarova C, Ferris J, Berry V, et al. (2004) Characterization of the G91del CRYBA1/3-crystallin protein: a cause of human inherited cataract. Hum Mol Genet 13: 945-953.
62. Ferrini W, Schorderet DF, Othenin-Girard P, Uffer S, Heon E, et al. (2004) CRYBA3/A1 gene mutation associated with suture-sparing autosomal dominant congenital nuclear cataract: a novel phenotype. Invest Ophthalmol Vis Sci 45: 1436-1441.
63. Qi Y, Jia H, Huang S, Lin H, Gu J, et al. (2004) A deletion mutation in the betaA1/A3 crystallin gene ( CRYBA1/A3) is associated with autosomal dominant congenital nuclear cataract in a Chinese family. Hum Genet 114: 192-197.
64. Lu S, Zhao C, Jiao H, Kere J, Tang X, et al. (2007) Two Chinese families with pulverulent congenital cataracts and deltaG91 CRYBA1 mutations. Mol Vis 13: 1154-1160.
65. Xu J, Wong C, Tan X, Jing H, Zhou G, et al. (2010) Decreasing the homodimer interaction: a common mechanism shared by the deltaG91 mutation and deamidation in betaA3-crystallin. Mol Vis 16: 438-444.
66. Billingsley G, Santhiya ST, Paterson AD, Ogata K, Wodak S, et al. (2006) CRYBA4, a novel human cataract gene, is also involved in microphthalmia. Am J Hum Genet 79: 702-709.
67. Zhou G, Zhou N, Hu S, Zhao L, Zhang C, et al.(2010) A missense mutation in CRYBA4 associated with congenital cataract and microcornea. Mol Vis 16: 1019-1024.
